# Inclusive Nutrition Care for LGBT+ Patients: Challenges and Opportunities for Dietitians—A Narrative Review

**DOI:** 10.3390/nu17203276

**Published:** 2025-10-18

**Authors:** Michał Czapla, Anthony Dissen

**Affiliations:** 1 Division of Scientific Research and Innovation in Emergency Medical Service, Department of Emergency Medical Service, Faculty of Nursing and Midwifery, Wroclaw Medical University, 51-618 Wroclaw, Poland; 2 Group of Research in Care (GRUPAC), Faculty of Health Sciences, University of La Rioja, 26006 Logroño, Spain; 3 School of Health Sciences, Stockton University, Galloway, NJ 08205, USA; anthony.dissen@stockton.edu

**Keywords:** dietetics, sexual and gender minorities, health equity, LGBT+ health

## Abstract

Nutrition is a cornerstone of public health, yet the unique nutrition needs and considerations of lesbian, gay, bisexual, transgender, and others (LGBT+) communities remain largely invisible in the field of dietetics. These populations face disproportionate burdens of obesity, eating disorders, body dysmorphia, metabolic risks, and food insecurity, often driven by stigma, minority stress, and structural inequities. This narrative review aimed to synthesize current evidence on nutrition-related disparities among LGBT+ populations and identify opportunities for dietitians to advance equity in care. A comprehensive search of PubMed, Scopus, and Web of Science was conducted for studies addressing diet quality, obesity, eating disorders, food insecurity, and metabolic health in sexual and gender minorities. Evidence indicates clear subgroup differences: lesbian and bisexual women are more likely to experience obesity and food insecurity; gay and bisexual men report lower BMI but greater body image concerns and disordered eating; transgender individuals face nutritional challenges linked to gender-affirming therapy and high rates of food insecurity; and people living with HIV encounter additional metabolic risks associated with treatment. Despite these findings, LGBT+ health remains rarely reflected in dietary guidelines or professional training. Embedding inclusivity into dietetic education and clinical encounters, adopting culturally competent and structurally aware practices, and tailoring interventions to subgroup-specific needs are key priorities. Inclusive, equity-driven, and person-centered nutrition care is essential to closing health gaps for LGBT+ populations and ensuring that every patient receives guidance that affirms their identity and lived experience.

## 1. Introduction

Nutrition is one of the cornerstone domains within the field of public health, shaping lifelong risk for obesity, cardiovascular disease, diabetes, and cancer [1,2,3,4,5,6]. Diet quality is now recognized as one of the most powerful determinants of population health worldwide, with poor nutrition contributing to an estimated 11 million premature deaths annually [7,8]. Beyond its biological impact, nutrition is also deeply embedded in social and cultural contexts, influencing how individuals access, choose, and experience food [9]. As public health shifts towards equity and person-centered care, it has become increasingly evident that nutrition cannot be addressed in a one-size-fits-all model [10]. Populations with unique social and health vulnerabilities, such as lesbian, gay, bisexual, transgender, and other sexual and gender minority (LGBT+) communities, require particular attention if equity in health outcomes is to be achieved [11].

LGBT+ populations experience persistent health inequities that extend across the life course. Minority stress, stigma, and discrimination shape social determinants of health, restricting access to safe housing, employment, and competent healthcare services [12,13,14,15]. These structural and interpersonal stressors are strongly associated with elevated rates of depression, anxiety, substance use, and suicide, but also drive behavioral patterns that affect diet and long-term metabolic risk [16,17,18]. Evidence consistently shows higher prevalence of obesity among lesbian and bisexual women, greater body image pressures and disordered eating among gay and bisexual men, and specific nutritional vulnerabilities among transgender individuals undergoing gender-affirming hormone therapy [19,20,21,22]. Such disparities underscore that the health of LGBT+ communities cannot be separated from the broader context of social exclusion, and they highlight the urgent need to integrate equity-focused approaches into clinical nutrition and dietetic practice.

Despite growing recognition of these inequities, nutrition remains an overlooked dimension of LGBT+ health. Most dietary guidelines worldwide, including the Dietary Guidelines for Americans and European recommendations, do not address the unique needs of sexual and gender minorities [23]. Existing evidence is fragmented, dominated by small cross-sectional studies and largely concentrated in North America and Western Europe, with scarce data from the Global South [24]. Research often aggregates LGBT+ individuals into a single category, obscuring subgroup-specific needs and reinforcing invisibility in clinical nutrition. For example, the metabolic changes associated with gender-affirming hormone therapy, or the elevated risk of food insecurity among sexual minority women and transgender people, are rarely incorporated into dietary recommendations [23,24,25,26]. This lack of tailored guidance leaves dietitians without evidence-based tools to provide equitable and inclusive nutrition care.

Dietitians and others within the allied health professionals occupy a central role in preventing and managing diet-related disease, providing counselling, education, and tailored nutrition interventions across diverse care settings [1,27]. Their close patient contact positions them to address inequities directly, yet most training curricula and clinical guidelines remain silent on the needs of LGBT+ individuals. Integrating cultural competence and inclusive practices into dietetic care is therefore essential if nutrition is to contribute meaningfully to reducing health disparities [28].

Recognizing this gap, our review seeks to synthesize existing evidence and to outline practical strategies for advancing equity in nutrition care for LGBT+ populations. This narrative review aims to examine nutrition-related health disparities among LGBT+ populations and to identify the challenges and opportunities for dietitians in delivering inclusive, culturally competent, and person-centered nutrition care.

## 2. Methods

This narrative review synthesizes current evidence on nutrition-related health disparities among LGBT+ populations. We searched the electronic databases PubMed, Scopus, and Web of Science between July–August 2025, using combinations of the following keywords e.g.: “LGBT”, “sexual minorities”, “gender minorities”, “diet”, “nutrition”, “food insecurity”, “eating disorders”, “obesity”, “metabolic risk”, and “hormone therapy”. Reference lists of relevant reviews and included articles were also screened to identify additional sources.

Inclusion criteria comprised peer-reviewed articles in English reporting original research or reviews on nutrition, dietary behaviors, or metabolic outcomes in LGBT+ populations. Eligible study designs included cross-sectional, cohort, case–control, and interventional studies, as well as narrative or systematic reviews. Exclusion criteria were case reports, conference abstracts and letters to the editor without original data. No restrictions were placed on study setting, geographic location, or population subgroup.

As this is a narrative review, we did not apply formal quality assessment tools or meta-analytic methods. Instead, we prioritized breadth of evidence to provide a comprehensive synthesis of available data and to highlight key gaps in research and practice.

## 3. Nutrition and Health Disparities in LGBT+ Populations

### 3.1. Diet Quality and Dietary Behaviors

Emerging evidence indicates that dietary behaviors vary significantly across LGBT+ populations, often reflecting the cumulative effects of minority stress, stigma, and structural barriers to food access. Nationally representative US data from NHANES show that gay and bisexual men have significantly higher overall dietary quality, measured by the Healthy Eating Index, compared with heterosexual men, whereas lesbian and bisexual women display no difference in overall scores despite their elevated risk of obesity and cardiometabolic disease [29]. These findings suggest that sexual orientation interacts with gender in shaping diet-related risk, with men and women facing distinct behavioral pathways.

Among sexual minority women, systematic reviews highlight inconsistent but concerning dietary trends. Some studies report lower vegetable intake and higher consumption of sugar-sweetened beverages compared with heterosexual women, while others find no differences once socioeconomic covariates are adjusted for. Importantly, bisexual women may consume more cereal fiber and whole grains than heterosexual peers, pointing to heterogeneity within the sexual minority population [30]. Nevertheless, sexual minority women remain disproportionately affected by obesity and related chronic conditions, indicating that dietary factors alone do not fully explain disparities [30,31].

Adolescents and young adults represent another group where disparities in diet quality are pronounced. Data from the Minnesota Student Survey and related analyses show that transgender and gender-nonconforming youth consume fewer fruits and milk and more fast food and soft drinks than cisgender peers, while also being more likely to skip meals due to socioeconomic hardship. These patterns were accompanied by higher rates of overweight and obesity and greater exposure to weight-based bullying. College-based surveys echo these findings, demonstrating that LGBT+ students report less frequent breakfast consumption, higher rates of meal skipping, and greater reliance on convenience foods compared with heterosexual students [32].

Taken together, these findings underscore that LGBT+ populations are not a monolith. While certain subgroups, such as gay and bisexual men, may demonstrate favorable diet quality scores, others—including sexual minority women and transgender youth—face compounded risks linked to both dietary behaviors and social determinants of health. Understanding these nuanced patterns is essential for developing inclusive nutritional guidelines and tailored interventions that address both biological and social drivers of disparities.

### 3.2. Obesity and Metabolic Health

Cardiometabolic inequities have been consistently documented among LGBT+ populations, although most evidence derives from North America and remains limited in Europe [33]. Obesity and related metabolic conditions are unevenly distributed across LGBT+ populations, reflecting both behavioral and structural determinants of health. Large-scale survey data from the United States indicate that lesbian and bisexual women are at consistently greater risk of overweight and obesity than their heterosexual peers [34]. Clinical data from Penn Medicine did not show differences between heterosexual and sexual minority women in the likelihood of receiving an obesity diagnosis or weight-loss recommendations. However, only about half of women with BMI ≥ 30 were appropriately diagnosed and counseled according to USPSTF recommendations, regardless of sexual orientation [31,35]. Overall, findings regarding obesity among lesbian and bisexual women remain inconsistent, likely reflecting methodological differences across studies and the complex interplay of social and behavioral determinants [34,36].

In contrast, gay and bisexual men tend to report lower mean BMI than heterosexual men, a difference often attributed to sociocultural pressures surrounding body image and leanness [23]. However, this apparent “protective” effect does not translate into reduced cardiometabolic risk, as disordered eating behaviors and high levels of stress can undermine metabolic health despite lower body weight [37]. These patterns underscore the need to move beyond BMI alone when assessing risk in sexual minority men.

Among transgender populations, gender-affirming hormone therapy introduces additional complexity. Recent longitudinal analyses demonstrate that feminizing hormone therapy is associated with increases in body fat and insulin resistance, while masculinizing regimens may raise hemoglobin and lipid levels [38]. These changes highlight the necessity of proactive metabolic monitoring and tailored dietary interventions for transgender individuals undergoing medical transition.

Taken together, evidence across subgroups indicates that obesity and metabolic health disparities in LGBT+ communities cannot be understood purely through diet or weight status. Instead, they are shaped by intersecting factors—minority stress, healthcare inequities, and hormone-related physiology that demand a more nuanced, inclusive approach to nutritional care and research.

### 3.3. Eating Disorders and Body Image

Eating disorders and body image concerns represent a major, yet underexplored, contributor to health disparities among LGBT+ populations. Evidence indicates that sexual and gender minority adolescents and adults experience higher rates of disordered eating behaviors including restrictive dieting, purging, and binge eating—than heterosexual and cisgender peers, with transgender youth facing particularly elevated risks [19]. Such disparities are closely linked to minority stress, weight-based victimization, and community-specific appearance ideals.

Additionally, patterns vary across subgroups. Gay and bisexual men consistently report heightened body dissatisfaction, self-criticism, and preoccupation with leanness and muscularity, reflecting cultural ideals reinforced by both mainstream and LGBT-specific media [39]. These pressures can translate into clinical eating disorders and maladaptive behaviors such as orthorexia nervosa, with recent cross-national data identifying links to Grindr use and PrEP practices [40]. By contrast, lesbian women often demonstrate more complex patterns: some studies suggest lower body uneasiness compared to heterosexual peers, yet higher rates of self-hate and binge eating, particularly in bisexual women [39].

Importantly, treatment pathways remain inequitable. A recent scoping review highlights that while conventional evidence-based therapies are broadly effective for LGBT+ adults with eating disorders, patients frequently describe discriminatory experiences in care settings, limiting engagement and recovery [41]. Tailored approaches that incorporate gender transition, community-specific support, and culturally competent care show promise but remain underdeveloped.

Taken together, these findings underscore that eating disorders in LGBT+ populations cannot be disentangled from broader social and cultural determinants. Addressing these disparities requires not only improved surveillance and subgroup-sensitive research but also inclusive treatment strategies that validate diverse body ideals and reduce structural stigma in clinical nutrition practice.

### 3.4. Food Insecurity and Intersecting Determinants of Health

Food insecurity has emerged as one of the most consistent nutrition-related disparities affecting LGBT+ populations. Nationally representative US data indicate that sexual minority adults, particularly bisexual individuals, face significantly higher odds of food insecurity compared with heterosexual peers [42]. This disparity is especially acute among younger adults and those living in unstable housing, highlighting how structural inequities compound dietary vulnerabilities.

Among students and emerging adults, food insecurity intersects with lower food literacy, disordered eating behaviors, and poor mental health [43]. A recent survey of LGBT+ college students found elevated rates of binge eating, restrictive dieting, and stress-related eating, all closely tied to experiences of food insecurity and minority stress [43]. Similar findings have been reported among transgender youth, who are more likely than cisgender peers to skip meals, rely on convenience foods, and report difficulty accessing nutritious options [23]. These behaviors both reflect and reinforce existing inequities in obesity, metabolic risk, and mental health.

Food insecurity rarely occurs in isolation. Its impact is magnified at the intersections of sexual orientation, gender identity, race/ethnicity, and HIV status. Evidence suggests that Black and Latinx sexual minority men face disproportionately higher rates of obesity and diabetes, while LGBT+ individuals living with HIV experience additional metabolic risks from antiretroviral therapy [33]. These findings underscore the importance of adopting an intersectional lens when analyzing nutrition inequities, as multiple layers of stigma and disadvantage converge to shape dietary health.

For dietitians and allied health professionals, addressing food insecurity requires moving beyond individual counselling to integrate advocacy, social support, and culturally competent care. Strategies that combine nutrition education with access to affordable, healthy foods, while acknowledging the lived realities of LGBT+ individuals, are essential to reducing disparities and advancing equity in nutrition security.

### 3.5. Additional Mental Health Considerations

Despite overall improvements in social acceptance of LGBT+ people and communities, there continue to be high rates of marginalization, stigma, and disparities experienced by members of this community [44]. And this has been shown to have direct impacts on mental and emotional wellbeing, particularly through oppression, discrimination, stigma, anti-trans and anti-queer policies, harassment, bullying, and other forms of microaggressions and macroaggressions [45,46,47,48]. Microaggressions are those statements, behaviors, actions, or thoughts that project oppression, discrimination, or prejudice towards members of a group, often in subtle, indirect, and even unconscious ways. Macroaggressions, on the other hand, are more systemic and institutionalized in nature, and work to oppress entire groups of people through structures, policies, and programs that create disparities and oppression. Being exposed to these forms of aggression can put one at an increased risk for loneliness and social exclusion, which, when coupled with other forms of minority stress, can significantly contribute to the development of poor mental health outcomes [48,49]. For example, microaggressions can take on a number of different forms, such as rude speech, insults, invalidations, and verbally or physically moving away from a person during communication. Macroaggressions can look like employment discrimination, denying access to care, or blocking access to housing. These factors contribute in significant ways to the mental health issues experienced by LGBT+ people. Research has suggested that LGBTQIA+ people use mental health services at higher rates when compared to heterosexual people, and experience higher rates of psychiatric disorders, substance abuse, and suicide [50].

## 4. Subgroup-Specific Considerations

Lesbian and bisexual women consistently exhibit higher prevalence of overweight and obesity compared with heterosexual women, a disparity observed across multiple epidemiological studies [19]. These differences have been linked to complex psychosocial and behavioral factors, including minority stress, coping strategies such as emotional eating, and divergent body image norms that may reduce pressure to pursue thinness compared with heterosexual peers. Evidence from clinical settings, however, remains mixed. Data from health systems with inclusive models of care, such as Penn Medicine, suggest that institutional context can mitigate disparities in obesity diagnosis and counseling observed elsewhere [35].

Dietary behaviors among sexual minority women also display considerable heterogeneity. Some studies report lower intake of fruits and vegetables and greater consumption of sugar-sweetened beverages compared with heterosexual women, whereas others show more favorable patterns, including higher cereal fiber and whole grain intake among bisexual women [43]. Food insecurity further compounds health risks: lesbian and bisexual women experience higher rates than heterosexual peers, which is strongly associated with binge eating, restrictive dieting, and adverse mental health outcomes [42].

Together, these findings indicate that lesbian and bisexual women face both elevated metabolic risks and structural vulnerabilities but also highlight the potential of inclusive healthcare environments to attenuate disparities. For dietitians, interventions must account for the diversity within this subgroup, integrating strategies that address food insecurity, minority stress, and culturally specific dietary norms.

Gay and bisexual men display a distinctive health profile marked by lower average BMI but greater vulnerability to disordered eating and body image concerns. A meta-analysis of over 93,000 UK participants found that gay men were significantly less likely to be overweight or obese compared with heterosexual men, yet more likely to be underweight, pointing to a double-edged pattern of risk [51]. These findings suggest that BMI alone provides an incomplete picture of nutritional and metabolic health in this group.

Body dissatisfaction is a central driver of these disparities. Systematic reviews confirm that gay and bisexual men experience higher rates of body dissatisfaction, greater “drive for thinness,” and elevated risk of eating disorders compared with heterosexual men [52]. Community and media dynamics amplify these pressures: cultural ideals within gay male subcultures often valorize both leanness and muscularity, creating contradictory appearance standards that foster internal conflict [53]. Exposure to dating apps such as Grindr further exacerbates these issues by promoting objectification and constant body comparison, which correlate with restrictive eating and orthorexic behaviors [40,54].

Despite these risks, community connectedness may serve as a protective factor. Data from cross-sectional studies show that stronger identification with and support from LGBT+ communities is associated with better sexual well-being and mitigated body dissatisfaction [55]. This highlights the dual role of community norms: while reinforcing appearance pressures, they may also provide resources for resilience and social support.

From a clinical perspective, these findings underscore the need to move beyond weight status in assessing health among gay and bisexual men. Dietitians and allied health professionals should actively screen for disordered eating behaviors and body image concerns, even in patients with “normal” or low BMI, and incorporate culturally sensitive strategies that address sociocultural stressors. Nutrition care for this population must integrate mental health support and challenge restrictive appearance ideals while fostering balanced approaches to diet and body composition.

Transgender and gender diverse individuals face some of the most profound nutrition-related inequities across sexual and gender minority groups. Epidemiological data indicate that lifetime prevalence of eating disorders is markedly elevated, reaching 10.5% among transgender men and 8.1% among transgender women, compared with 1.85% in cisgender populations [56]. Insurance-based analyses further show that over 2% of transgender patients carry an eating disorder diagnosis, with incidence peaking during early adolescence [57]. Body dysphoria, minority stress, and social exclusion are powerful drivers of restrictive dieting, binge eating, and compensatory behaviors in this group.

Gender-affirming hormone therapy (GAHT) introduces additional layers of nutritional complexity. Feminizing regimens are consistently associated with increases in fat mass, reduced insulin sensitivity, and higher prevalence of metabolic syndrome, while masculinizing therapy increases lean body mass but elevates hemoglobin, LDL cholesterol, and overall cardiovascular risk [58,59]. Longitudinal data suggest that risk for disordered eating may rise after initiation of feminizing therapy and decline in those on masculinizing regimens, underscoring the need for proactive dietary and psychological support throughout transition [60].

Beyond biomedical mechanisms, structural and social determinants profoundly shape nutrition in transgender populations. Surveys of transgender and gender-nonconforming students highlight disproportionately high levels of food insecurity, barriers to safe food access, and unmet nutritional needs within campus environments [61]. Systematic reviews confirm that food insecurity is highly prevalent among transgender individuals globally, exacerbating mental health challenges and disordered eating behaviors [21]. Intersectional disadvantages such as racial discrimination, housing instability, and HIV comorbidities further magnify nutritional vulnerabilities.

Taken together, these findings illustrate the multifaceted challenges facing transgender and gender diverse individuals, spanning metabolic shifts induced by GAHT, disproportionate burdens of eating disorders, and pervasive structural barriers to food security. For dietitians and allied professionals, inclusive nutrition care requires an interdisciplinary approach: integrating metabolic monitoring with endocrinology, tailoring dietary interventions to the physiological effects of hormone therapy, and addressing food insecurity as a structural determinant of health. The absence of formal dietary guidelines for transgender populations represents a critical gap that demands urgent attention in both research and practice.

Beyond subgroup-specific disparities, biomedical interventions such as HIV pre-exposure prophylaxis (PrEP) and antiretroviral therapy (ART) introduce additional nutrition-related considerations that cut across LGBT+ populations. Evidence from the iPrEx trial demonstrated that tenofovir disoproxil fumarate/emtricitabine (TDF/FTC) used as PrEP was not associated with adverse body composition changes; on the contrary, participants experienced smaller gains in fat mass and reductions in serum cholesterol compared with placebo, suggesting a relatively favorable metabolic profile [62]. These findings counter perceptions that PrEP uniformly drives weight gain, highlighting the importance of differentiating between preventive and therapeutic antiretroviral regimens.

In contrast, long-term ART in people living with HIV is consistently associated with dyslipidemia, insulin resistance, and weight gain, particularly in the context of integrase strand transfer inhibitors and protease inhibitors. Integrase strand transfer inhibitors are particularly associated with weight gain and metabolic complications, findings observed both in clinical trials and in real-world cohort studies [62,63,64]. These shifts elevate cardiovascular risk and necessitate proactive dietary counseling and lipid monitoring as part of routine care. Importantly, ART-related metabolic complications are not unique to heterosexual or LGBT+ populations but intersect with the higher HIV prevalence among gay, bisexual, and transgender individuals, thereby amplifying nutrition-related inequities.

Taken together, these data emphasize that PrEP and ART require distinct nutritional strategies: reassurance and monitoring in the case of TDF-based PrEP, and more intensive dietary and lifestyle interventions for long-term ART, especially regimens containing TAF or integrase inhibitors. For dietitians and allied professionals, engaging with these therapies as part of holistic, stigma-free nutrition care is essential to reducing cardiometabolic disparities across diverse sexual and gender minority groups.

## 5. Barriers to Inclusive Nutrition Care

Structural barriers remain a central driver of inequities in nutrition care for LGBT+ populations. Current dietary guidelines, including the Dietary Guidelines for Americans and European recommendations, are written as if nutritional needs were universal and do not acknowledge the specific risks and lived realities of sexual and gender minorities. This omission leaves dietitians without formal, evidence-based guidance to address disparities, reinforcing a one-size-fits-all model that fails to deliver equity [23,65]. Research gaps further exacerbate these shortcomings. The majority of available studies are small, cross-sectional, and geographically limited to North America, with scant representation from Europe, Latin America, Africa, or Asia [23,66]. These shortcomings are not confined to North America. In Europe, a recent cross-national study in Poland and Spain revealed significant gaps in LGBT+ clinical competence among health professionals, underlining the absence of standardized training across EU settings [17]. Narrative reviews further highlight that current dietary guidelines remain silent on LGBT+ health worldwide, reinforcing a one-size-fits-all approach and leaving practitioners without tailored guidance [23]. Moreover, sexual and gender minorities are frequently aggregated into a single category, obscuring subgroup-specific vulnerabilities such as food insecurity among bisexual women or the metabolic effects of hormone therapy in transgender individuals. This underrepresentation hampers the development of precise, population-specific dietary recommendations. Access barriers are compounded by systemic inequities in healthcare and education. Sexual and gender minority individuals are more likely to be uninsured, to experience discrimination in healthcare settings, and to report reduced trust in clinicians, all of which contribute to lower engagement with dietitians and preventive nutrition services [67]. At the same time, LGBT+ health remains poorly integrated into dietetics curricula worldwide, leaving many practitioners without the competence to deliver inclusive care [66]. Together, these systemic and structural barriers perpetuate invisibility in dietary guidance, restrict equitable access to nutrition services, and widen existing disparities in health outcomes for LGBT+ communities.

Barriers in clinical communication play a critical role in shaping the quality of nutrition care received by LGBT+ individuals. Many dietitians report limited training in culturally competent and inclusive language, leading to reliance on heteronormative assumptions during dietary assessments and counselling [17]. For example, intake forms and interviews often presume heterosexual partnerships or cisgender identities, which can alienate patients and hinder disclosure of relevant health behaviors [66]. Fear of stigma further undermines communication. Studies show that LGBT+ patients often withhold information about their sexual orientation, gender identity, or HIV/PrEP use because of anticipated discrimination, resulting in incomplete nutritional histories and missed opportunities for tailored interventions [68]. Even subtle microaggressions such as misgendering or assumptions about body goals can erode trust and discourage patients from continued engagement with dietetic services. Importantly, these barriers are not solely interpersonal but structural. Surveys of healthcare providers reveal persistent gaps in training on LGBT+ health, leaving clinicians unprepared to address specific concerns such as dietary implications of hormone therapy or disordered eating linked to body image pressures in gay and bisexual men [65]. Without systemic integration of inclusive practices, communication challenges perpetuate invisibility and reinforce inequities in nutrition care.

Educational deficits represent one of the most persistent barriers to inclusive nutrition care. Despite growing recognition of LGBT+ health inequities, sexual and gender diversity remains marginal in dietetics curricula worldwide. Qualitative research from Canada shows that dietetics education continues to reproduce cis- and heteronormative assumptions, with students reporting little or no exposure to LGBT+-specific case studies or community perspectives [66]. This lack of structured training leaves many graduates unprepared to navigate issues such as disordered eating linked to minority stress, or the nutritional implications of gender-affirming hormone therapy. Similar concerns are reflected across broader health professions [17]. Qualitative research in Canada has shown that dietetic training continues to reproduce cis- and heteronormative assumptions, while a recent scoping review confirmed the lack of standardized curricula and formalized guidance for LGBT+ health in dietetics education globally [65,66]. A national survey from India found that although most medical and nursing students expressed positive attitudes toward sexual and gender minority patients, they reported limited knowledge and low confidence in providing competent care [69]. These findings echo international reviews showing that LGBT+ health is poorly integrated into medical and allied health education, resulting in care that often defaults to heteronormative assumptions. The consequences of these educational gaps extend into clinical practice. Patients report experiences of microaggressions, erasure, or misgendering in dietetic encounters, often rooted in a lack of cultural competence and formal training [66]. Addressing these barriers requires more than ad hoc sensitivity workshops: it demands systemic curriculum reform, integration of queer pedagogy, and co-production of training modules with LGBT+ communities. Without such structural changes, the pipeline of future dietitians will remain ill-equipped to deliver equitable, person-centered nutrition care.

## 6. Opportunities for Dietitians and Future Directions

Dietitians and allied professionals are uniquely positioned to act as agents of change in advancing equity for LGBT+ communities. At the level of clinical practice, opportunities lie in reframing the nutrition encounter as a space of inclusion, trust, and recognition. This requires moving beyond generic dietary advice to approaches that respect diverse identities, lived experiences, and health trajectories. Simple shifts such as using inclusive language, acknowledging varied family and support structures, and explicitly addressing food insecurity can create profound improvements in patient engagement and outcomes.

Beyond biomedical considerations, culturally competent care for LGBT+ individuals requires acknowledging the social and psychological dimensions that shape nutrition behaviors and health outcomes. It involves recognizing how minority stress, stigma, and medical mistrust influence food choices, body image, and engagement with healthcare. Culturally competent practice moves beyond “treating everyone the same” toward care that is identity-affirming, trauma-informed, and responsive to the lived realities of diverse sexual and gender identities. This orientation allows dietitians to design interventions that not only address physiological needs but also restore agency, trust, and dignity within the nutrition encounter. These findings collectively highlight that nutrition-related disparities, including disordered eating patterns, are embedded within broader social and cultural determinants. For dietitians, acknowledging these upstream factors is essential for designing interventions that not only address physiological outcomes but also reduce stigma and support body acceptance across diverse identities. Likewise, addressing nutrition inequities requires adopting an intersectional lens that recognizes how multiple forms of stigma and disadvantage-related to gender identity, sexual orientation, socioeconomic status, and health conditions intersect to shape dietary behaviors and access to care.

Professional opportunities extend into advocacy. Dietitians can challenge discriminatory policies, ensure that food assistance programs account for the realities of LGBT+ lives, and contribute actively to the development of dietary guidelines that are explicitly inclusive. Interdisciplinary collaboration is also vital. Working alongside endocrinologists, infectious disease specialists, mental health providers, and community organizations will allow nutrition interventions to be integrated into the broader care continuum, particularly in relation to hormone therapy, HIV care, and mental health support.

Looking forward, the future of LGBT+ nutrition care must be anchored in education and research. Training programs for dietitians should embed inclusivity as a core competency, not an optional supplement, equipping graduates to address the unique challenges of minority stress, disordered eating, and metabolic consequences of gender-affirming care. Research agendas should shift from description to intervention: moving beyond small cross-sectional surveys towards longitudinal studies and trials that test practical, scalable nutrition strategies. Greater representation of LGBT+ populations in global nutrition science is essential, particularly from regions where evidence is currently absent.

In addition to cultural and intersectional awareness, developing structural competence is essential for advancing equitable nutrition care. Structural competence emphasizes understanding how social policies, institutional practices, and systemic inequities shape nutrition behaviors, healthcare access, and health outcomes for LGBT+ populations. By cultivating this competence, dietitians can identify and challenge structural barriers such as discriminatory food policies, gaps in insurance coverage, or the absence of inclusive clinical protocols thereby promoting systemic rather than solely individual change.

Finally, dietitians must embrace their role not only as clinicians but also as advocates, educators, and researchers committed to dismantling inequities. The opportunity is to build a future where nutrition care is not merely “inclusive” in rhetoric but substantively equitable in practice where every patient, regardless of identity, can access guidance that recognizes and affirms their realities. Without such action, the field will continue to reproduce existing disparities. With it, dietetics can emerge as a leader in equity-driven public Health.

## 7. Conclusions

Nutrition-related disparities among LGBT+ populations are evident across diet quality, obesity, eating disorders, and food insecurity, and are shaped by stigma, minority stress, and structural inequities. Despite these challenges, LGBT+ communities remain largely invisible in dietary guidelines, research agendas, and professional training, leaving dietitians without the tools to deliver equitable care. To address these gaps, dietetics must move beyond a one-size-fits-all model. Embedding inclusivity in education, clinical practice, and research, while strengthening collaboration with other disciplines and LGBT+ communities, will be essential. Only by integrating equity at every level can nutrition care contribute meaningfully to closing health gaps and advancing public health.

## Data Availability

Not applicable.

## Abbreviations

The following abbreviations are used in this manuscript:

ART	Antiretroviral Therapy
BMI	Body Mass Index
GAHT	Gender-Affirming Hormone Therapy
HIV	Human Immunodeficiency Virus
LGBT+	Lesbian, Gay, Bisexual, Transgender, and other sexual and gender minorities
PrEP	Pre-Exposure Prophylaxis
